# Comparative Enzymatic and Gene Expression Responses in Wheat to DON- and NIV-Producing *Fusarium* Species

**DOI:** 10.3390/biology14081063

**Published:** 2025-08-16

**Authors:** Gabriela da Rocha Lemos Mendes, Paulo Cesar Pazdiora, Vivian Ebeling Viana, Leandro José Dallagnol, Laura Christina Calgaro, Glacy Jaqueline da Silva, Emerson Medeiros Del Ponte, Antônio Costa de Oliveira

**Affiliations:** 1 Centro de Genômica e Fitomelhoramento, Faculdade de Agronomia Eliseu Maciel, Universidade Federal de Pelotas, Pelotas 96010-900, Brazil; vivianebelingviana@hotmail.com; 2 Departamento de Fitossanidade, Faculdade de Agronomia Eliseu Maciel, Universidade Federal de Pelotas, Pelotas 96010-900, Brazil; paulo.pazdiora@yahoo.com.br (P.C.P.); leandro.dallagnol@ufpel.edu.br (L.J.D.); 3 Departamento de Biotecnologia, Universidade Paranaense, Umuarama 87502-210, Brazil; laura.calgaro@edu.unipar.br (L.C.C.); glacyjaqueline@prof.unipar.br (G.J.d.S.); 4 Departamento de Fitopatologia, Universidade Federal de Viçosa, Viçosa 36570-000, Brazil; delponte@ufv.br

**Keywords:** *Fusarium*–wheat interaction, ROS, enzymes, genes, FHB, plant biotic stress

## Abstract

*Fusarium* head blight (FHB) is a major threat to wheat production that is caused by toxigenic species of the *Fusarium graminearum* complex. This study aimed to investigate the biochemical and molecular defense responses of Brazilian wheat genotypes (BRS 194, BRS Parrudo, and Frontana) with contrasting FHB susceptibilities following inoculation with *F. graminearum* (deoxynivalenol producer) and *F. meridionale* (nivalenol producer). Temporal patterns of antioxidant enzymes, defense-related enzymes, and gene expression (*ABC-Transporter* and *Ca^2+^-ATPase*) were analyzed from 12 to 96 h after inoculation. The ANOVA results revealed significant effects of genotypes, inoculation, and time after inoculation on most of the evaluated enzymatic activities. Frontana exhibited high basal activity for most enzymes, and after inoculation, the enzyme activity was higher than in other genotypes. BRS 194 presented delayed and fragmented activation patterns, particularly under DON-producing pathogen infection. According to the transcriptome results, inoculation with the NIV-producing pathogen upregulated both genes, reaching up to an 18-fold increase. BRS 194 showed an upregulated transcript pattern from the early hours after inoculation. Frontana showed increased transcript levels, reaching 12-fold, under DON-producing pathogen infection. These findings show that biochemical and molecular responses varied depending on genotype and the chemotype of the *Fusarium* isolate, highlighting the importance of early, coordinated defense activation in FHB resistance.

## Simple Summary

Wheat, one of the most important food crops, is seriously affected by *Fusarium* head blight, a fungal disease damaging grain yield and quality. In this study, we analyzed three Brazilian wheat genotypes that react differently to this disease. Our goal was to understand how these plants defend themselves by looking at the activity of enzymes and the behavior of specific genes. Frontana showed a strong and early defense, activating protective mechanisms quickly. BRS 194 reacted later and less effectively, which may explain why it is more susceptible to the disease. We also found that the response of the plants varied depending on the type of fungus that caused the infection. These results provide valuable information that can help develop better strategies for breeding resistant wheat plants. Stronger resistance will help farmers reduce losses, improve food production, and decrease the need for chemical treatments, benefiting both the agricultural sector and society.

## 1. Introduction

*Fusarium* head blight (FHB) is a major fungal disease of small grains worldwide, particularly wheat and barley, leading to significant yield losses and reduced grain quality [1]. The primary causal agent is *Fusarium graminearum* anamorph (syn.) of *Gibberella zeae* (Schwein.) Petch (1936) (Ascomycota: Hypocreales), although other members of the *F. graminearum* species complex (FGSC) also contribute to disease outbreaks in specific regions [2]. Beyond yield impact, these pathogens threaten food safety due to the production of mycotoxins such as deoxynivalenol (DON) and nivalenol (NIV), which contaminate grains and pose health risks to humans and animals [3,4]. In Brazil, the DON-producing *F. graminearum* predominates, followed by the NIV-producing *Fusarium meridionale* T. Aoki, Kistler, Geiser & ODonnell (2004), with both mycotoxins frequently detected in commercial wheat [5,6].

FHB resistance in wheat is quantitative and involves multiple defense layers, making genetic improvement and disease management challenging [7]. Resistance is typically categorized into five types: Type I (resistance to initial infection), Type II (resistance to fungal spread within the spike), Type III (resistance to mycotoxin accumulation), Type IV (resistance to kernel infection), and Type V (tolerance to yield loss and physiological damage) [8,9,10]. The effectiveness of these resistance types is influenced by both environmental conditions and the aggressiveness of the infecting *Fusarium* chemotype [11].

Following pathogen recognition, wheat plants initiate a cascade of biochemical and molecular responses aimed at limiting infection and minimizing damage. Early defense reactions include the rapid production of reactive oxygen species (ROS), calcium influx, activation of mitogen-activated protein kinase (MAPK) signaling cascades, and cell wall reinforcement [12,13,14]. ROS play a dual role as antimicrobial agents and as signaling molecules that regulate defense gene expression. However, uncontrolled ROS accumulation can cause oxidative damage and trigger host cell death, making ROS homeostasis essential for effective resistance [11,14,15]. This balance is maintained through both non-enzymatic antioxidants such as glutathione and enzymatic systems including superoxide dismutase (SOD), catalase (CAT), peroxidase (POX), and ascorbate peroxidase (APX) [16,17]. Transcriptomic studies have shown that FHB-resistant wheat genotypes exhibit enrichment of oxidative stress-related pathways, highlighting the importance of ROS regulation in resistance [18].

In addition to oxidative stress control, other biochemical pathways contribute to wheat defense against FHB. Enzymes such as phenylalanine ammonia-lyase (PAL) and lipoxygenase (LOX) participate in the biosynthesis of phenolic compounds and oxylipins, strengthening structural barriers and amplifying defense signaling [11,19]. Additionally, pathogenesis-related (PR) proteins, including chitinase (CHI) and β-1,3-glucanase (GLU), degrade fungal cell wall components, restricting pathogen growth and contributing primarily to Type II and Type IV resistance [9].

At the molecular level, the regulation of specific defense-related genes further enhances the plant’s ability to cope with infection. *ABC transporters* and *UDP-glycosyltransferases* are associated with Type III resistance by facilitating mycotoxin detoxification and reducing the toxic impact of trichothecenes [18,20,21]. Calcium signaling genes, such as *Ca^2+^-ATPases*, play a central role in early signal transduction and ROS regulation, potentially contributing to both Type I and Type II resistance [21,22]. Collectively, the integration of these biochemical and molecular responses defines the plant’s capacity to limit disease progression and mitigate damage.

Previous studies conducted in Brazilian wheat germplasm have identified common defense mechanisms activated in response to DON- and NIV-producing *Fusarium* species, but with significant variability in resistance levels among genotypes [7]. The present study aims to investigate the temporal dynamics of key biochemical and molecular defense responses in three Brazilian wheat genotypes with contrasting susceptibility to FHB following infection with DON-producing *F. graminearum* and NIV-producing *F. meridionale*. Specifically, we evaluated the activities of antioxidant and defense-related enzymes, along with the expression of detoxification- and signaling-related genes, to better understand their roles in FHB resistance under subtropical conditions. Recent reviews highlight the increasing use of integrative multi-omics approaches to dissect complex plant–pathogen interactions and identify key genes and pathways involved in *Fusarium* head blight resistance in wheat [23].

## 2. Materials and Methods

### 2.1. Wheat Genotypes

The wheat genotypes used in this study, BRS 194, BRS Parrudo, and Frontana, were specifically chosen based on their distinct responses to FHB in terms of disease severity, as previously reported [7]. The genotype Frontana has been identified as a valuable source of resistance in breeding programs [24], while BRS Parrudo exhibits novel plant ideotype and is moderately resistant to FHB [25]. Conversely, BRS 194 represents a susceptible genotype, which is widely used in FHB assays [7].

### 2.2. Fungal Isolates and Inoculum Production

Two fungal isolates were chosen to represent the two major phylogenetic species associated with FHB in Brazil, namely *F. graminearum* (Schwabe) (hereafter Fgra) and *F. meridionale* (Aoki, Kistler, Geiser & ODonnell) (hereafter Fmer) [26]. The selection was based on their known aggressiveness and toxigenic potential established in previous studies. The isolates were chemotyped as 15-acetyldeoxynivalenol (15-ADON) for *F. graminearum* and nivalenol (NIV) for *F. meridionale* [7,26,27]. These isolates were deposited at the Mycological Collection of Lavras (Coleção Micológica de Lavras—CML) with the codes CML 3344 (Fmer isolate) and CML 3066 (Fgra isolate). The inoculum was produced in *Spezieller Nährstoffarmer Agar* (SNA) media [6] and consisted of a macroconidial suspension with a concentration of 10^5^ spores mL^−1^.

### 2.3. Inoculation Procedures and Plant Sampling

For each wheat genotype, ten plastic pots with a capacity of four liters and containing autoclaved soil were sown with ten seeds, and five plants were grown in each pot. The pots were then kept in a greenhouse from seeding to flowering stage under controlled conditions of 25 ± 5 °C and 60% relative humidity. Inoculation was performed at full flowering stage using the central-spikelet method, where 10 µL of macroconidia suspension of either Fgra or Fmer was gently applied using a micropipette inside each floret of a spikelet located in the central portion of the spike. As a control, plants were mock inoculated with distilled water using the same method as for the fungal plant inoculation. Following inoculation, plants were placed in a moist chamber maintained at 25 ± 2 °C and 95 ± 2% relative humidity for 48 h. Tissue samples from inoculated and mock-inoculated florets (three spikelets above and below the inoculation point) were harvested at 12, 24, 48, 72, and 96 h after inoculation (abbreviated as HAI). The minimum sample size consisted of three random ears per genotype, inoculation, and sampling point, which were collected and pooled for biochemical and molecular assays. The samples were randomly collected and were immediately wrapped in aluminum foil, frozen in liquid nitrogen, and stored at −80 °C until analysis.

### 2.4. Determination of Enzymatic Activities

The determination of the following enzymes was performed: catalase (CAT, EC 1.11.1.6), superoxide dismutase (SOD, EC 1.15.1.1), peroxidase (POX, EC 1.11.1.7), polyphenol oxidase (PPO, EC 1.10.3.1), phenylalanine ammonia-lyase (PAL, EC 4.3.1.5), lipoxygenase (LOX, EC 1.13.11.12), chitinase (CHI, EC 3.2.1.14), and β-1,3-glucanase (GLU, EC 3.2.1.39). The crude enzyme extract was obtained from the pool of either inoculated or mock-inoculated spikelet tissues macerated until becoming a fine powder in liquid nitrogen. A 0.5 g aliquot of the powdered tissue was mixed with 40 mg of polyvinylpolypyrrolidone (PVPP), and then homogenized in 3 mL of potassium phosphate buffer (pH 6.5, 100 mM) containing 1 mM phenylmethylsulphonyl fluoride (PMSF) and 1 mM ethylenediaminetetraacetic acid (EDTA), as described [28]. The homogenate was centrifuged at 12,000× *g* for 15 min at 4 °C, and the supernatant was collected and immediately used for enzymatic activity assays.

The activity of CAT was determined by quantifying the decomposition of hydrogen peroxide (H_2_O_2_) (Merck, Pinheiros/SP, Brazil), measuring the decrease in absorbance at 240 nm [29] and expressed as micromoles of H_2_O_2_ degraded min^−1^ mg^−1^ of protein. The SOD activity was estimated based on the colorimetric quantification of the nitroblue tetrazolium (NBT) (Sigma-Aldrich, Jurubatuba/SP, Brazil) photoreduction, according to [30]. The unit of SOD (U) corresponded to the amount of enzyme capable of inhibiting 50% of NBT photoreduction [28]. The POX activity and PPO activity were determined based on the colorimetric quantification of pyrogallol (Sigma-Aldrich, São Paulo/SP, Brazil) oxidation and expressed as micromoles of purpurogallin produced min^−1^ mg^−1^ of protein using an extinction coefficient of 2.47 mM^−1^ cm^−1^. assayed by pyrogallol (Sigma-Aldrich, Jurubatuba/SP, Brazil) oxidation according to [31,32]. The absorbance was measured in a spectrophotometer (model UV-UM51, Bel Engineering ^®^, Monza/MB, Italy) at 420 nm. The PAL activity was determined by colorimetric quantification of trans-cinnamic acid formed from phenylalanine (Sigma-Aldrich, Jurubatuba/SP, Brazil), which was measured in a spectrophotometer at 290 nm (model UV-UM51, Bel Engineering srl^®^, Monza/MB, Italy). The molar extinction coefficient of 10^4^ mM^−1^ cm^−1^ [33] was used to calculate PAL activity, which was expressed in moles of transcinnamic acid produced per min^−1^ mg^−1^ of protein [34].

The LOX activity was determined by quantification of linolenic acid hydroperoxide quantified in a spectrophotometer (model UV-UM51, Bel Engineering srl^®^, Monza/MB, Italy) at 234 nm [35]. The extinction coefficient of 25,000 M^−1^ cm^−1^ was used to calculate the LOX activity and expressed as micromoles min^−1^ mg^−1^ protein. The CHI activity was assayed following the colorimetric determination of p-nitrophenyl cleaved from a chitin-analogous substrate, p-nitrophenyl-β-DN, N′- diacetylchitobiose (PNP) (Sigma-Aldrich, São Paulo/SP, Brazil) at 410 nm [36]. The molar extinction coefficient (7 × 10^4^ mM^−1^ cm^−1^) used to calculate CHI activity was expressed as micromoles of p-nitrophenyl produced per min^−1^ mg^−1^ of protein. To evaluate the GLU activity, the DNS (3,5-dinitrosalicylic acid) (Sigma-Aldrich, Jurubatuba/SP, Brazil) method was used measuring the reducing sugars released by the β-1,3-glucanase action. The absorbance was measured at 540 nm and GLU activity was expressed in absorbance units min^−1^ mg^−1^ of protein [37]. The soluble protein concentrations of the extracts were measured by the standard Bradford method, using bovine serum albumin as the standard protein wavelength (595 nm) [38].

### 2.5. Quantitative Real-Time PCR (RT-qPCR) Analysis

For RNA extraction, freeze-dried samples were macerated in liquid nitrogen. Total RNA was extracted using the Trizol reagent (Invitrogen, Carlsbad, CA, USA), according to manufacturer’s instructions. The RNA was precipitated and diluted in RNase-free water and the quantity and quality were evaluated by spectrophotometer (NanoVue Plus, GE HealthCare, Chicago, IL, USA) and agarose gel (2%) electrophoresis. cDNA was synthesized with 2.0 µg of total RNA, using SuperScript III First Strand Synthesis System (Invitrogen, Waltham, MA, USA) according to manufacturer’s instructions.

The expression profile of the *ABC-Transporter* and *Ca^2+^-ATPase* genes were analyzed. Both genes are related to wheat defense responses to *Fusarium* [22,39]. PCR primers were validated by amplification curve analyses employing a pool of cDNAs from all tested conditions, at four distinct dilutions (1:1, 1:5, 1:25 and 1:125). All primers used in this study showed efficiency between 1.8–2.2 and the specificity and absence of primer dimers were checked by dissociation curves. Gene expression analysis (qPCR) was performed using ABI PRISM 7500 Fast (Applied Biosystems, Foster, CA, USA). Each reaction contained 10 μL Fast SYBR Green Master Mix (Thermo Fisher Scientific, Waltham, MA, USA), 1 μL cDNA (diluted 1:25), 7 μL of RNase-free water, 10 μM of both forward and reverse primer in a final volume of 20 μL. The reactions started with a denaturation step at 95 °C for 10 min, followed by 40 cycles of 15 s at 95 °C and 60 s at 60 °C, finalized by the dissociation curve with denaturation at 95 °C for 15 s, cooling at 60 °C for 60 s. After the final PCR cycle, a melting curve analysis was conducted to determine the reaction specificity. A negative, no template control (NTC), was used to confirm the absence of genomic DNA.

Target gene expression was quantified using the comparative 2^−ΔΔCt^ method [40]. The expression of each target gene is presented as fold change normalized to the reference genes ubiquitin and RNA-Helicase [39,41] and relative to the mock-inoculated (untreated control sample). The primers used for RT-qPCR expression analyses was shown in Appendix Table A1.

### 2.6. Experimental Design and Data Analysis

The experiment was arranged in a completely randomized design, a 3 × 3 × 5 factorial experimental, consisted of the combination of the three wheat genotypes (Frontana, BRS Parrudo and BRS 194), three inoculations [two fungal species (Fgra and Fmer) and mock inoculated] and five times of plant tissue sampling (12, 24, 48, 72 and 96 HAI). Mock inoculated plants were included in the experiment as negative control. Each experimental unit consisted of a pool of at least three inoculated spikes from different plants. The homoscedasticity and normality of residuals were evaluated. Data from biochemical analyses were subjected to ANOVA and the significant effects were compared by Tukey’s test (*p* ≤ 0.05), to verify the interaction effects, using STATISTICA (version 13). Data from gene expressions were expressed as relative mRNA abundance in a color diagram using Multi Experiment Viewer software version 4.9 (Multi Experiment Viewer 4.9, TIGR, Rockville, MD, USA) [42].

## 3. Results

The ANOVA results (shown in Appendix Table A2) revealed significant effects of genotypes (F1), inoculation (F2), and time after inoculation (F3) on most of the evaluated enzymatic activities. Subsequent pairwise comparisons using Tukey’s HSD tested for different means at each time point (see Table S1), and the *p*-value for all comparisons were also calculated and are presented in Table S2. The wheat genotype had a significant influence on all enzymes, indicating intrinsic differences in the basal and induced defense responses among genotypes. Inoculation effects, i.e., different chemotypes and mock-inoculated, were significant for POX, PPO, SOD, and CHI, suggesting a pathogen-induced modulation of these enzymes. Time after inoculation significantly affected the activity of all enzymes, highlighting the temporal dynamics of the defense response. The enzymatic results were organized by functional categories according to their roles in plant defense.

### 3.1. Antioxidant Enzymes Activity in Wheat-Fusarium Interaction

The activities of antioxidant enzymes varied among wheat genotypes during infection by Fgra and Fmer, indicating differential oxidative stress responses associated with resistance and susceptibility. The enzymatic profiles are described below for each genotype.

**Figure 1:**
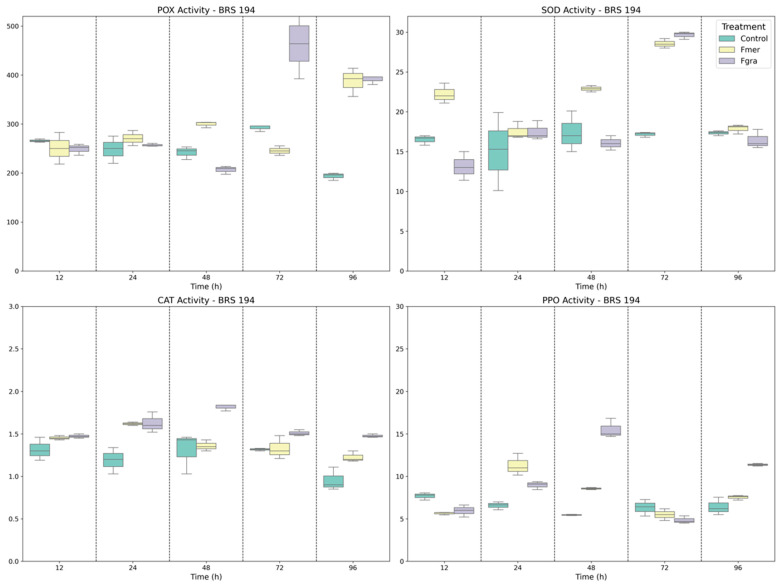
Specific activities of POX, SOD, CAT, and PPO enzymes in BRS 194 wheat genotype following mock inoculation (control) or inoculation with Fmer and Fgra at 12, 24, 48, 72, and 96 h after inoculation (HAI).

In BRS 194, a susceptible genotype, a dynamic enzymatic response to both pathogens were observed throughout the infection timeline (Figure 1). SOD activity increased significantly as early as 12 HAI with Fmer, suggesting an immediate oxidative burst in response to infection. In contrast, SOD induction in response to Fgra was delayed, peaking only at 72 HAI, although by this time point no significant difference was observed between the two inoculation treatments (Fgra and Fmer). CAT activity was markedly elevated in inoculated plants from 24 HAI onwards, particularly under Fgra infection, which triggered the highest CAT levels at both 48 and 96 HAI. POX activity was strongly induced, showing a significant increase beginning at 48 HAI, with a pronounced peak at 72 HAI under Fgra inoculation—the highest POX activity recorded across all genotypes and time points. PPO activity displayed a biphasic response, with an initial peak at 24 HAI following Fmer inoculation and subsequent increases at 48 and 96 HAI in response to Fgra, consistent with the delayed establishment of cell wall-based defenses.

**Figure 2:**
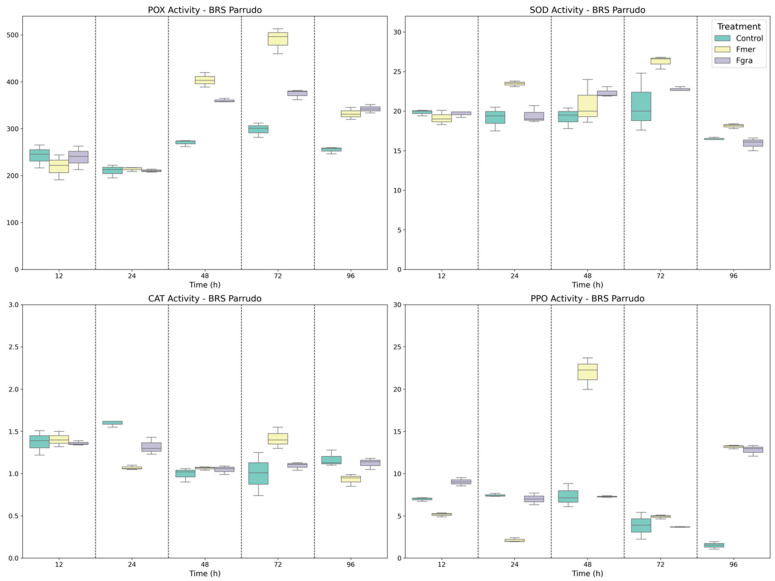
Specific activity of POX, SOD, CAT, and PPO enzymes in BRS Parrudo wheat genotype following mock inoculation (control) or inoculation with Fmer and Fgra at 12, 24, 48, 72, and 96 h after inoculation (HAI).

In the moderately resistant genotype BRS Parrudo, the enzymatic response was characterized by more gradual and temporally spaced increases in antioxidant activity (Figure 2). SOD activity rose at 24 and 72 HAI in response to Fmer, with no significant activation observed under Fgra infection. CAT activity remained relatively stable across most time points, except for a significant increase at 72 HAI upon Fmer inoculation, suggesting a delayed but specific ROS-scavenging response. POX activity was moderately induced at 48 and 72 HAI by Fmer, indicating activation of the apoplastic ROS detoxification system, although the magnitude of response was lower than that observed in BRS 194. PPO activity increased significantly only at 96 HAI in response to both pathogens, although Fmer treatment resulted in an earlier elevation at 48 HAI. As PPO is associated with phenolic oxidation and the reinforcement of defense barriers, this delayed but enhanced activity may contribute to the partial resistance observed in this genotype.

**Figure 3:**
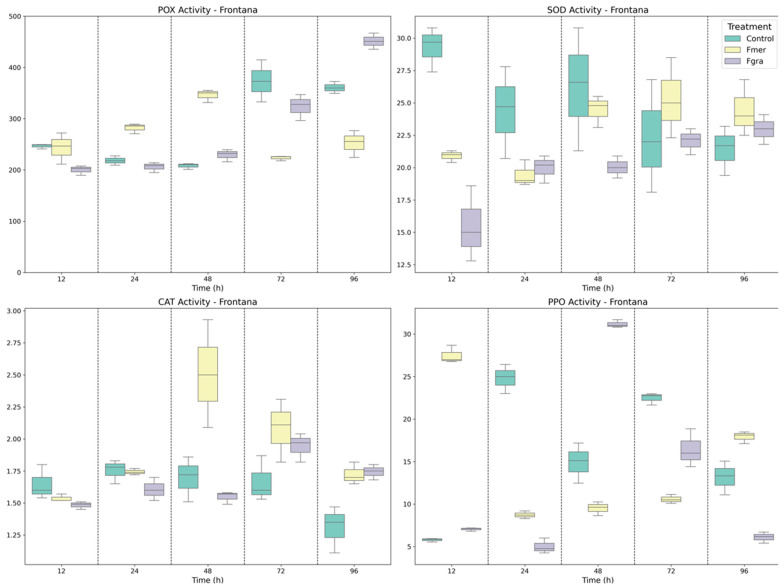
Specific activity of POX, SOD, CAT, and PPO enzymes in Frontana wheat genotype following mock inoculation (control) or inoculation with Fmer and Fgra at 12, 24, 48, 72, and 96 h after inoculation (HAI).

Finally, Frontana, a genotype widely recognized for its resistance, a constitutive and early activated antioxidant system was evident (Figure 3) Notably, SOD activity was highest under mock-inoculated conditions, demonstrating a strong basal oxidative defense capacity, which may contribute to the genotype’s resistance to *Fusarium*. No significant changes in SOD activity were observed upon infection, suggesting that basal levels were sufficient to buffer the initial oxidative stress. CAT activity was significantly upregulated at 48 HAI in response to Fmer, representing the most pronounced CAT response among all genotypes and inoculation treatments, and indicating robust control of H_2_O_2_ accumulation. POX activity was induced earlier in Frontana than in the other genotypes, with a significant increase at 24 HAI under Fmer inoculation, indicative of a rapid apoplastic defense mechanism. PPO activity was also elevated under basal conditions, and Fmer inoculation resulted in activity peaks at 12 and 96 HAI. In contrast, Fgra triggered a sharp and significant PPO increase at 48 HAI, the highest PPO activity detected across all genotypes, reinforcing its role in late-stage defense through phenolic compound oxidation and quinone formation.

### 3.2. Defense-Related Enzymes in Phenolic Biosynthesis, Signaling, and Fungal Cell Wall Degradation

**Figure 4:**
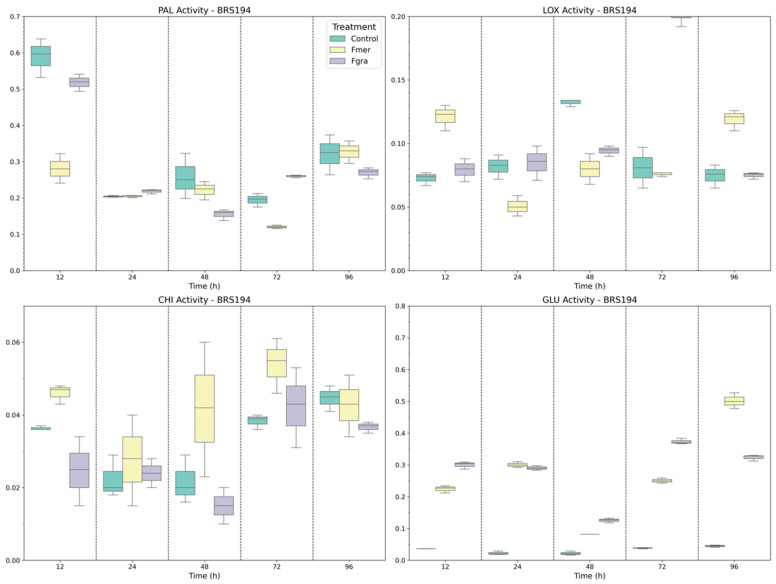
Specific activity of PAL, LOX, CHI, and GLU enzymes in BRS194 wheat genotype following mock inoculation (control) or inoculation with Fmer and Fgra at 12, 24, 48, 72, and 96 h after inoculation (HAI).

In addition to the antioxidant enzymes, other defense-related enzymes, including PAL, LOX, CHI, and GLU, play critical roles in plant-pathogen interactions. In BRS 194 (Figure 4), distinct temporal patterns were observed for all enzymes in response to both pathogens. PAL activity was lower or similar to the mock-inoculated plants at most time points, except at 72 HAI, where Fgra inoculation resulted in significantly higher activity compared to both mock and Fmer-inoculated plants. LOX activity exhibited a more stable pattern during the infection process, with slight fluctuations. A higher LOX activity was detected at 12 and 96 HAI in Fmer-inoculated plants compared to Fgra and mock-inoculated plants, suggesting a modest activation of oxylipin pathways.

Differences in CHI activity between treatments were detected only at 12 HAI, with Fmer inoculation inducing a two-fold increase compared to Fgra. At later time points, no significant differences were observed, suggesting that CHI activation in BRS 194 was limited and not strongly sustained during the later stages of infection. The inoculation with both Fgra and Fmer significantly increased GLU activity compared to the mock-inoculated plants at all time points. Notably, Fgra-inoculated plants exhibited significantly higher GLU activity at 12, 48, and 72 HAI, suggesting a stronger and more sustained activation by the DON-producing isolate during the intermediate stages of infection. In contrast, Fmer inoculation induced a later response, with a pronounced peak in GLU activity observed at 96 HAI.

**Figure 5:**
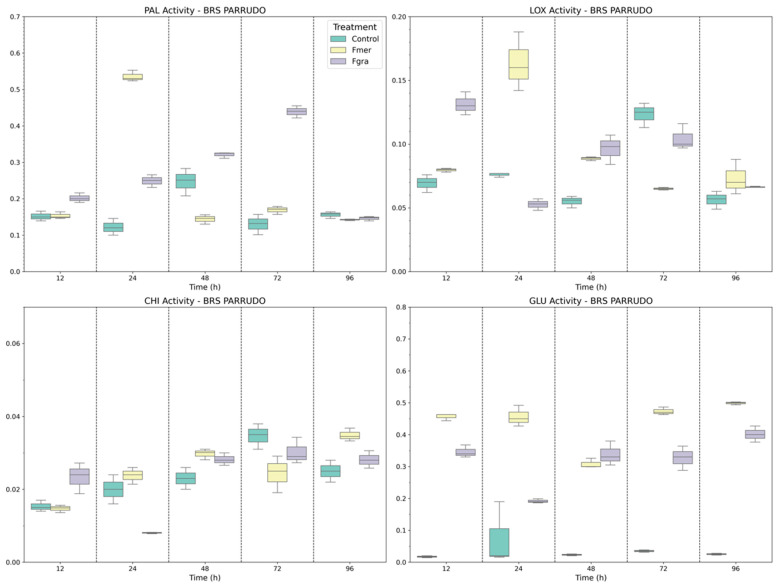
Specific activity of PAL, LOX, CHI, and GLU enzymes in BRS Parrudo wheat genotype following mock inoculation (control) or inoculation with Fmer and Fgra at 12, 24, 48, 72, and 96 h after inoculation (HAI).

In BRS Parrudo, distinct and time-dependent enzymatic responses were observed in when comparing pathogens. PAL activity showed a gradual increase during Fgra inoculation, with significant peaks at 48 and 72 HAI (Figure 5). In contrast, Fmer-induced PAL activity was only higher than that of mock-inoculated plants at 24 HAI. By 96 HAI, no significant differences among treatments were observed. LOX activity showed an early induction in response to both Fgra and Fmer inoculations, peaking at 12 and 24 HAI, respectively. After these time points, enzyme activity declined, and by 72 and 96 HAI, no significant differences were observed between inoculated and mock-inoculated plants.

CHI activity, as observed in BRS 194, exhibited modest variations across treatments. While Fgra inoculation resulted in increased CHI activity at 12 HAI, Fmer inoculation lead to a later response, only at 96 HAI. GLU activity was also induced following Fgra and Fmer inoculations, showing increased levels from 12 HAI onward compared to mock-inoculated plants (Figure 5). Notably, Fmer-inoculated plants exhibited the highest GLU activity starting at 12 HAI and maintained it until 96 HAI. The only time point without significant differences between Fgra- and Fmer-inoculated plants was at 48 HAI.

**Figure 6:**
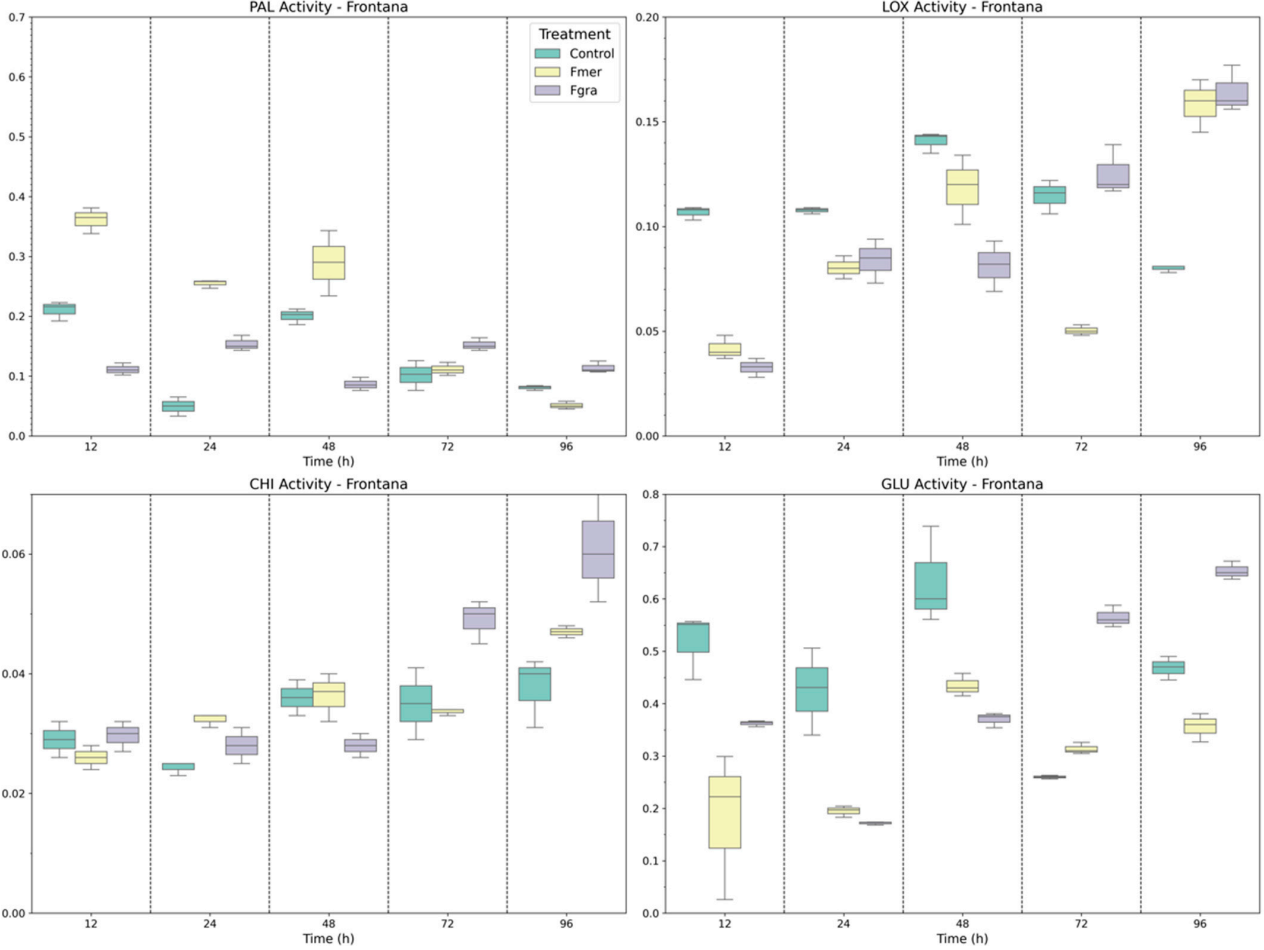
Specific activity of PAL, LOX, CHI, and GLU enzymes in Frontana wheat genotype following mock inoculation (control) or inoculation with Fmer and Fgra at 12, 24, 48, 72, and 96 h after inoculation (HAI).

In Frontana, the enzymes related to phenolic synthesis and fungal cell wall degradation displayed distinct temporal patterns when inoculated and mock-inoculated plants were compared (Figure 6). In general, the basal activity of these enzymes was higher when compared to inoculated plants. PAL activity exhibited an early and pronounced induction at 12 HAI in response to Fmer inoculation, with elevated levels maintained up to 48 HAI. Conversely, Fgra-inoculated plants maintained consistently lower and relatively stable PAL activity throughout the time points of infection. The basal activity of LOX, indicated by mock-inoculated plants, was surprisingly higher for all time points evaluated, except at 96 HAI when the inoculated plants displayed a two-fold increase in the LOX activity. When Fgra and Fmer inoculations were compared, differences were seen only at 48 and 72 HAI.

As shown in Figure 6, CHI activity presented modest variations at earlier time points (12–48 HAI). However, a significant increase was observed from 72 HAI onwards, especially in Fgra-inoculated plants, which reached the highest CHI levels at 96 HAI, followed by Fmer and mock-inoculated plants. This suggests a delayed but strong CHI-mediated defense activation primarily triggered by Fgra. Similar to LOX and CHI, GLU activity exhibited high basal levels in mock-inoculated plants. Following Fgra inoculation, a continuous increase in GLU activity was observed from 24 HAI onwards, reaching peak levels at 72 and 96 HAI.

### 3.3. Gene Expression

Differences in transcript accumulation were observed across the five evaluated time points for the different fungal species. The genes exhibited distinct expression patterns in response to each pathogen, and therefore, the results were presented separately for each fungal species.

**Figure 7:**
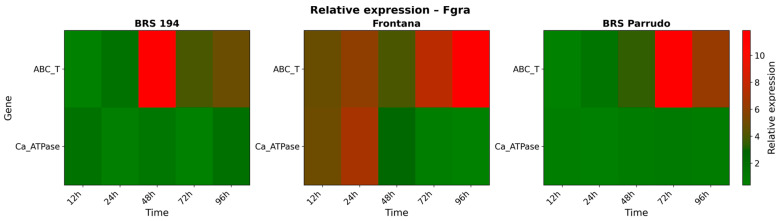
Relative transcript accumulation of *ABC-Transporter* and *Ca^2+^-ATPase* genes in BRS 194, Frontana, and BRS Parrudo inoculated with Fgra at 12, 24, 48, 72, and 96 h after inoculation (HAI). Gene expression was normalized to the respective mock-inoculated controls at each time point. Relative mRNA abundance is represented on a scale from 0 to 12 using the MultiExperiment Viewer (TIGR MeV) software, v. 4.9.

A strong induction of *ABC-Transporter* gene expression was observed in all genotypes, but with different timing and intensity (Figure 7). In BRS 194, the highest expression level occurred at 48 HAI, showing a pronounced peak (over 10-fold increase relative to mock-inoculated), followed by a gradual decline at later time points (72 and 96 HAI). Frontana showed a more gradual and sustained induction, with expression progressively increasing from 24 HAI onwards, reaching the highest levels at 72 and 96 HAI, 3- and 5-fold increases in transcript levels, respectively. In BRS Parrudo, the induction was delayed compared to the other genotypes, with moderate expression at 48 HAI and a more pronounced accumulation at 72 HAI, reaching over a 10-fold increase relative to mock-inoculated plants.

The *Ca^2+^-ATPase* gene exhibited relatively stable expression across all time points in BRS 194 and BRS Parrudo, with both genotypes showing a modest increase, reaching just over a 2-fold change compared to mock-inoculated plants (Figure 7). On the other hand, Frontana displayed higher transcript levels from as early as 12 HAI, with a peak expression observed at 24 HAI (2.8-fold increase).

**Figure 8:**
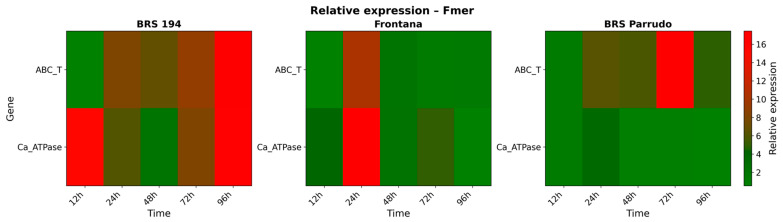
Relative transcript accumulation of *ABCTransporter* and *Ca^2+^-ATPase* genes in BRS 194, Frontana, and BRS Parrudo inoculated with Fmer at 12, 24, 48, 72, and 96 h after inoculation (HAI). Gene expression was normalized to the respective mock-inoculated controls at each time point. Relative mRNA abundance is represented on a scale from 0 to 18 using the Multi Experiment Viewer (TIGR MeV) software, v. 4.9.

Following Fmer inoculation, both genes exhibited distinct transcriptional responses across genotypes and time points, with notable increases in relative expression levels, as shown in Figure 8. Among the genotypes, BRS 194 showed the most pronounced response, with consistently higher transcript accumulation at all evaluated time points. Induction of the *ABCTransporter* gene in BRS 194 was detected from 24 HAI onwards, reaching a peak of over 15-fold expression at 96 HAI compared to mock-inoculated plants. On the other hand, Frontana displayed a transient transcriptional activation, with a notable peak at 24 HAI, reaching approximately 12-fold induction, followed by a return to lower levels at later time points. For BRS Parrudo, a pattern similar to BRS 194 was observed, with early gene induction at 24 HAI (over 8-fold increase), followed by a peak expression at 72 HAI, reaching more than 16-fold relative to the mock-inoculated plants.

Similar to the pattern observed for the *ABCTransporter* gene, the transcriptional analysis of the *Ca^2+^-ATPase* gene revealed a genotype-dependent response following Fmer inoculation. BRS 194 exhibited the strongest induction, with a marked peak in transcript levels as early as 12 HAI, followed by a second increase at 96 HAI, reaching over 16-fold expression compared to mock-inoculated plants.

In Frontana, a sharp and transient induction was detected at 24 HAI, also surpassing a 16-fold increase, but this was followed by a rapid decline in expression at subsequent time points. On the other hand, BRS Parrudo maintained relatively low and stable transcript levels throughout the infection period, with expression remaining close to 2-fold compared to the mock-inoculated plants at all evaluated time points.

## 4. Discussion

In this study, the effects of inoculating wheat genotypes with a DON-producing *F. graminearum* and a NIV-producing *F. meridionale* were examined, with a focus on molecular and biochemical changes over a period of 96 h. Although several studies have investigated individual components of the wheat–*Fusarium* interaction, there is still limited understanding of the coordinated temporal activation of biochemical and molecular defense mechanisms. This type of analysis is important for understanding the levels of enzymes present in cells during post-translational regulation. Such integrative approaches help to clarify the multilayered nature of FHB resistance, which involves diverse and interacting defense pathways, as highlighted in recently proposed models [10].

Our previous study indicated that BRS Parrudo had higher levels of resistance compared to BRS 194 [6]. The present study reveals distinct patterns of biochemical and molecular responses depending on genotype, pathogen species, and time post-inoculation.

In the highly susceptible genotype BRS 194, defense responses appeared fragmented and delayed. Although a strong early induction of *Ca^2+^-ATPase* was observed following Fmer inoculation (12 HAI), this was not accompanied by sustained enzymatic defense. The delayed and fluctuating activities of GLU and CHI, particularly under Fgra inoculation, suggest an ineffective mobilization of structural defenses. Similarly, the antioxidant enzymes CAT, POD, and PFO showed limited or late activation, with only moderate SOD induction around 48–72 HAI. Of particular interest is the sharp rise in *ABCTransporter* expression at 96 HAI with Fgra, which may reflect a late attempt at detoxification in response to DON accumulation. This genotypic profile suggests weak regulatory coordination and insufficient priming for early and robust defense.

A delayed activation of SOD may contribute to the level of susceptibility, since SOD represents the first line of defense against the toxic effects of elevated ROS levels [43], and its delayed induction in response to *F. graminearum* species might reflect a compromised initial defense signaling.

The results reveal that Frontana possesses a high basal antioxidant capacity and, following Fmer and Fgra inoculations, showed a rapid and sustained increase in SOD, CAT, and PFO activities, particularly between 24 and 72 HAI. This is in agreement with its previous classification as a source of Type II resistance, i.e., resistance to fungal spread within the spike [44]. The early and consistently high activities of CHI and GLU further support this classification, as these enzymes contribute to cell wall reinforcement, synthesis of antimicrobial compounds, and degradation of fungal structures—processes directly associated with Type II resistance [12,14].

Supporting this biochemical profile, Frontana has been genetically characterized as carrying key Type II resistance QTLs. Recently, a meta-analysis study reported that this genotype harbors two major Type II FHB resistance QTLs on chromosome 5A, spanning the 205–524 Mb interval. These QTLs overlap with the physical location of the *Qfhb.ndwp-5A* locus, further reinforcing the genetic basis for its robust defense response [45].

Frontana exhibited an early and strong upregulation of *ABC-Transporter* and *Ca^2+^*-*ATPase* genes during Fgra infection. However, the enzymatic activity showed a more subtle and regulated response, possibly avoiding an excessive energy-consuming reaction. This behavior may be related to the basal resistance strategy characteristic of this genotype.

*ATP-binding cassette* (ABC) transporters are transmembrane proteins that use the energy from ATP hydrolysis to transport substances across the cell membrane. Studies indicate the possible association of these proteins with removal of fungal toxins (and other xenobiotics) from the cytoplasm, preventing or minimizing cytotoxicity [39,46]. In wheat, genes of the ABC family are candidates for enhancing FHB resistance and reducing DON accumulation in the grains [9].

The precise timing of genetic and biochemical responses is essential for an effective response to pathogen attack and to trigger a priming physiological state. Our results support Frontana as a model of coordinated Type II resistance to FHB and suggest improved outcomes in terms of mycotoxin accumulation, likely due to increased *ABCTransporter* expression.

Previously, the *calcium-transporting ATPase* gene was related to the defense process, mainly in the first hours after infection, since this gene is a major regulator of intracellular Ca^2+^ concentrations and acts to support proper cell signaling [22].

BRS Parrudo shows an intermediate pattern, with pathogen-specific and temporally regulated activation of defense enzymes. The earlier activation observed under Fmer inoculation compared with Fgra across genotypes further supports the hypothesis that these pathogens elicit distinct oxidative stress signaling pathways in wheat. GLU and CHI activities were induced early and maintained at high levels, particularly under Fgra inoculation, indicating rapid cell wall reinforcement. The early activation of these enzymes is crucial for the basal defense response. Pathogenesis-related (PR) proteins, such as PR-2 (β-1,3-glucanase) and PR-3 (chitinase), are typically upregulated following pathogen infection and tend to accumulate at higher levels during the early stages of the defense response [9].

PAL and LOX activities also increased at intermediate stages, suggesting activation of salicylic acid and jasmonic acid pathways [22]. Of note, *ABC-Transporter* expression peaked significantly at 72 HAI with Fgra. The minimal variation in *Ca^2+^-ATPase* expression observed in BRS Parrudo may indicate a more tightly regulated intracellular calcium flux. However, further studies—particularly at earlier time points, such as within the first 3 h after inoculation—are needed to clarify the role of *Ca^2+^-ATPase* in wheat–*Fusarium* interactions.

Previous studies have shown that the expression pattern of *Ca^2+^-ATPase* can vary depending on the host genotype. Upon infection with *F. graminearum*, this gene was reported to be up- and downregulated in susceptible and resistant genotypes, respectively [20]. In our study, *Ca^2+^-ATPase* was upregulated across all genotypes and time points evaluated; however, the magnitude of expression was strongly influenced by the chemotype of the infecting *Fusarium* species. This reinforces the idea that resistance involves complex and multilayered responses, including calcium signaling and detoxifying pathways. Recent multi-omics investigations have emphasized the role of such regulatory components alongside the phenylpropanoid pathway, UGTs, and GSTs as key contributors to FHB defense mechanisms in wheat [23]. These results are consistent with QTL-based studies that identified UDP-glucosyltransferases and peroxidases as contributors to DON detoxification and oxidative stress control in FHB-resistant wheat lines [47].

## 5. Conclusions

Overall, our findings demonstrate that resistant genotypes such as Frontana possess a high basal antioxidant capacity and rapidly activate defense-related enzymes, particularly in response to *F. meridionale* infection. In contrast, the susceptible genotype BRS 194 exhibits delayed yet pronounced oxidative responses, which may represent a compensating rather than an effective protective mechanism. BRS Parrudo presents an intermediate profile, with defense activation that is both pathogen-specific and temporally regulated. The earlier responses observed under *F. meridionale* inoculation across all genotypes further support the notion that distinct chemotypes of the *F. graminearum* species complex elicit different signaling dynamics in wheat.

These results underscore the importance of temporal coordination in plant defense. The differential regulation of defense enzymes and signaling genes highlights the complexity of the wheat–*Fusarium* interaction and suggests that effective resistance is associated not only with the magnitude but also with the timing and integration of defense responses. This integrative biochemical and molecular analysis identifies potential defense markers and provides valuable insights for breeding programs aiming to enhance FHB resistance by targeting key timing and signaling components.

## Data Availability

The original contributions presented in this study are included in the article. Further inquiries can be directed to the corresponding authors.

## Supplementary Materials

The following supporting information can be downloaded at: https://www.mdpi.com/article/10.3390/biology14081063/s1, Table S1: Results of Tukey’s HSD multiple comparisons for the enzymes peroxidase (POX), superoxide dismutase (SOD), catalase (CAT), polyphenol oxidase (PPO), lipoxygenase (LOX), phenylalanine ammonia-lyase (PAL), chitinase (CHI), and β-1,3-glucanase (GLU), at each time point (12 h, 24 h, 48 h, 72 h, 96 h) for each genotype (Frontana, BRS 194, BRS Parrudo). Table S2: *p*-values from Tukey’s HSD pairwise comparisons for all treatment combinations at each evaluation time point for the enzymatic activities assessed.

## Abbreviations

The following abbreviations are used in this manuscript:

HAI	Hours after inoculation
SOD	Superoxide dismutase
CAT	Catalase
POX	Peroxidase
PPO	Polyphenol oxidase
PAL	Phenylalanine ammonia-lyase
GLU	β-1,3-glucanases
QUI	Chitinase
LOX	Lipoxygenase
PR protein	Pathogenesis-related protein
Fgra	*Fusarium graminearum* (DON producer)
Fmer	*Fusarium meridionale* (NIV producer)
DON	Deoxynivalenol
NIV	Nivalenol
FGSC	*F. graminearum* species complex
ROS	Reactive oxygen species
FHB	*Fusarium* head blight

## Appendix A

**Table A1:** Sequence of primers used in the qPCR assay.

Transcript	Forward Primer	Sequence ID GeneBank	Reverse Primer	Author
*ABC-Transporter*	GCCAACGAATA GATCCTCCA	XM_044491856.1	ACCACCTTCAGCCT TTTCCT	[38]
*Ca^2+^*-*ATPase*	TCCTGATGAAAAGGGCAACC	XM_044539437.1	GCACAGCACCAGAT ACAAATGC	[46]
*Ubiquitin*	CCCTGGAGGTG GAGTCATCTGA	AK446922.1	GCGGCCATCCTCAA GCTGCTTA	[40]
*RNA-helicase*	GCACAGGGAAT CGTCAAAGT	XM_044542377.1	CACACATAGCTTCA TTCGTTCA	[38]

**Table A2:** Analysis of variance of the effects of wheat genotypes (F1), inoculation (F2), time points after inoculation (F3), and interactions on the variables catalase (CAT), superoxide dismutase (SOD), peroxidase (POX), polyphenol oxidase (PPO), phenylalanine ammonia-lyase (PAL), lipoxygenase (LOX), chitinase (CHI), and β-1,3-glucanase (GLU).

Source of Variation	DF	F Values							
		POX	PPO	CAT	SOD	LOX	PAL	GLU	QUI
F1	2	8.3 *	406.3 *	151.1 *	75.6 *	20 *	212.5 *	200 *	0 *
F2	2	8.2 *	4.9 *	0.9 ^ns^	24.2 *	1 ^ns^	0.6 ^ns^	0 ^ns^	15 *
F1 × F2	4	6.5 *	31.7 *	15.9 *	18.8 *	20 *	62.5 *	130 *	15 *
F3	4	31.1 *	53.9 *	10.4 *	53.7 *	20 *	50 *	50 *	50 *
F1 × F3	8	22.7 *	21.4 *	13.5 *	3.5 *	30 *	68.7 *	60 *	5 *
F2 × F3	8	13.1 *	63.7 *	7.9 *	3.2 *	40 *	47.5 *	30 *	4.5 *
F1 × F2 × F3	16	23.4 *	88.3 *	7.4 *	10.7 *	30 *	22.5 *	40 *	4.5 *

DF = degrees of freedom; * significant at *p* ≤ 0.05; ^ns^ = not significant.
