# Supplementary material for: Comparative Enzymatic and Gene Expression Responses in Wheat to DON- and NIV-Producing Fusarium Species
Source: Biology (Basel). 2025 Aug 16;14(8):1063. doi: 10.3390/biology14081063 (PMC12383778; doi:10.3390/biology14081063)
Supplement: Supplementary file 1 [file biology-14-01063-s001.zip › biology-3781126 Table S1.pdf]

Supplementary material

Table S1. Results of Tukey's HSD multiple comparisons for the enzymes peroxidase (POX), superoxide dismutase (SOD), catalase (CAT), polyphenol oxidase (PPO), lipoxygenase (LOX), phenylalanine ammonia-lyase (PAL), chitinase (CHI), and β-1,3-glucanase (GLU), at each time point (12 h, 24 h, 48 h, 72 h, 96 h) for each genotype (Frontana, BRS 194, BRS Parrudo). Cell values are the mean enzyme activities for each treatment (Control, Fgra, Fmer). Lowercase letters denote homogeneous groups within each column (time + enzyme + genotype): treatments sharing the same letter do not differ significantly ($p > 0.05$), whereas different letters indicate significant differences ($p \leq 0.05$). Asterisks (*) next to a time point mark columns in which at least one pairwise comparison was statistically significant.

**FRONTANA**																				
	POX					SOD					CAT					PPO				
	12 h	24 h *	48 h *	72 h *	96 h *	12 h *	24 h	48 h	72 h	96 h	12 h	24 h	48 h *	72 h	96 h *	12 h *	24 h *	48 h *	72 h *	96 h *
Control	246.56 a	218.30 b	207.96 b	373.53 a	360.63 b	29.30 a	24.40 a	26.23 a	22.30 a	21.43 a	1.64 a	1.75 a	1.69 b	1.66 a	1.31 b	5.78 b	24.82 a	14.91 b	22.47 a	13.16 b
Fgra	200.13 a	205.90 b	229.33 b	323.53 b	451.16 a	15.46 c	19.96 a	20.03 a	22.06 a	22.96 a	1.48 a	1.60 a	1.54 b	1.94 a	1.74 a	7.04 b	5.00 c	31.16 a	16.42 b	6.09 c
Fmer	243.40 a	281.93 a	354.77 a	223.40 b	252.23 c	20.90 b	19.43 a	24.46 a	25.26 a	24.43 a	1.53 a	1.74 a	2.50 a	2.08 a	1.72 a	27.48 a	8.72 b	9.50 c	10.57 c	17.91 a
**FRONTANA**																				
	LOX					PAL					QUI					GLU				
	12 h *	24 h *	48 h *	72 h *	96 h *	12 h *	24 h *	48 h *	72 h *	96 h *	12 h	24 h *	48 h *	72 h *	96 h *	12 h *	24 h *	48 h *	72 h *	96 h *
Control	0.10 a	0.10 a	0.14 a	0.11 a	0.08 b	0.21 b	0.04 c	0.20 b	0.10 b	0.08 b	0.02 a	0.02 b	0.03 a	0.03 b	0.03 b	0.51 a	0.42 a	0.63 a	0.25 c	0.46 b
Fgra	0.03 b	0.08 b	0.08 b	0.12 a	0.16 a	0.11 c	0.15 b	0.08 c	0.15 a	0.11 a	0.02 a	0.02 b	0.02 b	0.04 a	0.06 a	0.36 a	0.17 b	0.37 b	0.56 a	0.65 a
Fmer	0.04 b	0.08 b	0.11 a	0.05 b	0.15 a	0.36 a	0.25 a	0.28 a	0.11 b	0.05 c	0.02 a	0.03 a	0.03 a	0.03 b	0.04 b	0.18 b	0.19 b	0.43 b	0.31 b	0.35 c
**BRS 194**																				
	POX					SOD					CAT					PPO				
	12 h	24 h	48 h *	72 h *	96 h *	12 h *	24 h	48 h *	72 h *	96 h	12 h	24 h *	48 h *	72 h	96 h *	12 h *	24 h *	48 h *	72 h	96 h *
Control	265.90 a	248.36 a	241.96 b	292.36 b	193.63 b	16.50 b	15.10 a	17.36 b	17.16 b	17.33 a	1.31 a	1.19 b	1.30 b	1.31 a	0.95 c	7.68 a	6.56 c	5.45 c	6.34 a	6.41 b
Fgra	249.00 a	257.36 a	206.73 c	464.46 a	391.13 a	13.13 c	17.50 a	16.06 b	29.63 a	16.43 a	1.47 a	1.62 a	1.81 a	1.51 a	1.47 a	5.95 b	8.97 b	15.51 a	4.84 a	11.37 a
Fmer	250.30 a	270.90 a	299.56 a	245.46 b	387.56 a	22.23 a	17.53 a	22.90 a	28.56 a	17.86 a	1.45 a	1.62 a	1.36 b	1.33 a	1.22 b	5.64 b	11.29 a	8.56 b	5.49 a	7.53 b
**BRS 194**																				
	LOX					PAL					QUI					GLU				

	12 h *	24 h *	48 h *	72 h *	96 h *	12 h *	24 h *	48 h *	72 h *	96 h	12 h *	24 h	48 h	72 h	96 h	12 h *	24 h *	48 h *	72 h *	96 h *
Control	0.07 b	0.08 a	0.13 a	0.08 b	0.07 b	0.58 a	0.20 b	0.25 a	0.19 b	0.32 a	0.03 b	0.02 a	0.02 a	0.03 a	0.04 a	0.03 c	0.02 b	0.02 c	0.03 c	0.04 c
Fgra	0.07 b	0.08 a	0.09 b	0.20 a	0.07 b	0.51 a	0.21 a	0.15 b	0.26 a	0.26 a	0.02 b	0.02 a	0.01 a	0.04 a	0.03 a	0.30 a	0.29 a	0.12 a	0.37 a	0.32 b
Fmer	0.12 a	0.05 b	0.08 b	0.07 b	0.11 a	0.28 b	0.20 b	0.22 a	0.12 c	0.32 a	0.04 a	0.02 a	0.04 a	0.05 a	0.04 a	0.22 b	0.30 a	0.08 b	0.25 b	0.50 a

BRS PARRUDO

	POX					SOD					CAT					PPO				
	12 h	24 h	48 h *	72 h *	96 h *	12 h	24 h *	48 h	72 h	96 h *	12 h	24 h *	48 h	72 h	96 h *	12 h *	24 h *	48 h *	72 h	96 h *
Control	242.40 a	210.16 a	270.03 c	298.06 c	254.76 b	19.83 a	19.13 b	19.23 a	20.80 a	16.50 b	1.37 a	1.59 a	0.99 a	1.00 a	1.17 a	6.98 b	7.44 a	7.34 b	3.85 a	1.50 b
Fgra	238.80 a	210.36 a	359.93 b	374.23 b	342.33 a	19.66 a	19.46 b	22.33 a	22.76 a	15.90 b	1.36 a	1.32 b	1.04 a	1.09 a	1.12 a	9.02 a	7.01 a	7.28 b	3.68 a	12.79 a
Fmer	216.00 a	214.33 a	403.80 a	489.80 a	332.06 a	19.13 a	23.46 a	20.86 a	26.23 a	18.13 a	1.40 a	1.07 c	1.06 a	1.41 a	0.93 b	5.14 c	2.11 b	21.98 a	4.89 a	13.18 a

BRS PARRUDO

	LOX					PAL					QUI					GLU				
	12 h *	24 h *	48 h *	72 h *	96 h	12 h *	24 h *	48 h *	72 h *	96 h	12 h *	24 h *	48 h *	72 h	96 h *	12 h *	24 h *	48 h *	72 h *	96 h *
Control	0.06 b	0.07 b	0.05 b	0.12 a	0.05 a	0.15 b	0.12 c	0.24 b	0.13 b	0.15 a	0.01 b	0.02 a	0.02 a	0.03 a	0.02 b	0.01 c	0.07 b	0.02 b	0.03 c	0.02 c
Fgra	0.13 a	0.05 b	0.09 a	0.10 a	0.06 a	0.20 a	0.24 b	0.32 a	0.43 a	0.14 a	0.02 a	0.00 b	0.02 b	0.03 a	0.02 b	0.34 b	0.19 b	0.33 a	0.32 b	0.40 b
Fmer	0.07 b	0.16 a	0.08 a	0.06 b	0.07 a	0.15 b	0.53 a	0.14 c	0.16 b	0.14 a	0.01 b	0.02 a	0.02 b	0.03 a	0.03 a	0.45 a	0.45 a	0.30 a	0.47 a	0.49 a
