# Supplementary material for: Comparative Enzymatic and Gene Expression Responses in Wheat to DON- and NIV-Producing Fusarium Species
Source: Biology (Basel). 2025 Aug 16;14(8):1063. doi: 10.3390/biology14081063 (PMC12383778; doi:10.3390/biology14081063)
Supplement: Supplementary file 1 [file biology-14-01063-s001.zip › biology-3781126 Table S2.pdf]

Supplementary material

Table S2. *p*-values from Tukey's HSD pairwise comparisons for all treatment combinations at each evaluation time point for the enzymatic activities assessed. Values correspond to the statistical comparisons presented in Table S1.

Enzyme	Time	FRONTANA			BRS Parrudo			BRS 194		
		Control vs Fgra	Control vs Fmer	Fgra vs Fmer	Control vs Fgra	Control vs Fmer	Fgra vs Fmer	Control vs Fgra	Control vs Fmer	Fgra vs Fmer
POX	12h	0.0513	0.9766	0.0664	0.9836	0.5345	0.6302	0.5801	0.6249	0.9965
POX	24h	0.323	0.0005	0.0002	0.9996	0.8290	0.8432	0.8366	0.3569	0.6494
POX	48h	0.1085	0.0000	0.0000	0.0001	0.0000	0.0043	0.0102	0.0008	0.0001
POX	72h	0.1556	0.0015	0.0112	0.0066	0.0000	0.0008	0.0060	0.4178	0.0018
POX	96h	0.0027	0.0010	0.0000	0.0001	0.0002	0.4546	0.0000	0.0000	0.9687
SOD	12h	0.0003	0.0049	0.0352	0.9414	0.4000	0.5678	0.0470	0.0044	0.0004
SOD	24h	0.1102	0.0759	0.9540	0.9270	0.0068	0.0100	0.6124	0.6047	0.9999
SOD	48h	0.0860	0.7442	0.2231	0.1754	0.5519	0.6136	0.6024	0.0125	0.0045
SOD	72h	0.9955	0.5187	0.4718	0.5442	0.0505	0.2049	0.0000	0.0000	0.0768
SOD	96h	0.5803	0.1839	0.6055	0.3857	0.0189	0.0043	0.4054	0.7050	0.1486
CAT	12h	0.1113	0.3020	0.7200	0.9855	0.9137	0.8400	0.1208	0.1776	0.9513
CAT	24h	0.1223	0.9859	0.1501	0.0047	0.0001	0.0078	0.0082	0.0089	0.9972
CAT	48h	0.7753	0.0222	0.0103	0.5314	0.3617	0.9342	0.0122	0.8968	0.0200
CAT	72h	0.2458	0.0793	0.6644	0.7796	0.0502	0.1192	0.0640	0.9787	0.0821
CAT	96h	0.0116	0.0143	0.9782	0.7617	0.0235	0.0557	0.0009	0.0216	0.0314
PPO	12h	0.1041	0.0000	0.0000	0.0008	0.0015	0.0000	0.0110	0.0051	0.7358
PPO	24h	0.0000	0.0000	0.0173	0.5002	0.0000	0.0000	0.0307	0.0012	0.0352
PPO	48h	0.0000	0.0096	0.0000	0.9980	0.0000	0.0000	0.0000	0.0032	0.0000
PPO	72h	0.0045	0.0001	0.0053	0.9726	0.4054	0.3122	0.1051	0.3991	0.5602
PPO	96h	0.0012	0.0092	0.0001	0.0000	0.0000	0.5924	0.0002	0.1557	0.0007
LOX	12h	0.0000	0.0000	0.1152	0.0001	0.2205	0.0002	0.6179	0.0010	0.0022
LOX	24h	0.0136	0.0069	0.7998	0.1721	0.0006	0.0001	0.9368	0.0260	0.0175
LOX	48h	0.0024	0.1403	0.0227	0.0010	0.0031	0.4477	0.0020	0.0003	0.1250
LOX	72h	0.3365	0.0002	0.0001	0.0642	0.0003	0.0025	0.0000	0.8475	0.0000
LOX	96h	0.0001	0.0002	0.7430	0.4108	0.1337	0.6512	0.9982	0.0007	0.0007
PAL	12h	0.0009	0.0001	0.0000	0.0056	0.9847	0.0066	0.1681	0.0002	0.0010
PAL	24h	0.0001	0.0000	0.0001	0.0004	0.0000	0.0000	0.0187	0.9953	0.0208
PAL	48h	0.0130	0.0381	0.0007	0.0205	0.0041	0.0002	0.0452	0.5493	0.1805

PAL	72h	0.0247	0.7734	0.0571	0.0000	0.1153	0.0000	0.0009	0.0005	0.0000
PAL	96h	0.0030	0.0054	0.0001	0.2244	0.0935	0.7781	0.2894	0.9769	0.2245
CHI	12h	0.9451	0.3777	0.2574	0.0242	0.9548	0.0174	0.1015	0.1755	0.0090
CHI	24h	0.1356	0.0061	0.0801	0.0037	0.2654	0.0009	0.9674	0.7240	0.8546
CHI	48h	0.0462	0.9907	0.0394	0.0590	0.0210	0.6796	0.7739	0.1719	0.0709
CHI	72h	0.0130	0.9157	0.0085	0.4321	0.0510	0.2720	0.8109	0.1076	0.2384
CHI	96h	0.0108	0.2598	0.0852	0.3276	0.0062	0.0346	0.2419	0.8941	0.4148
GLU	12h	0.1609	0.0086	0.1050	0.0000	0.0000	0.0001	0.0000	0.0000	0.0001
GLU	24h	0.0016	0.0027	0.8346	0.1231	0.0006	0.0042	0.0000	0.0000	0.2900
GLU	48h	0.0029	0.0115	0.3963	0.0000	0.0000	0.3380	0.0000	0.0000	0.0002
GLU	72h	0.0000	0.0072	0.0000	0.0000	0.0000	0.0006	0.0000	0.0000	0.0000
GLU	96h	0.0001	0.0022	0.0000	0.0000	0.0000	0.0005	0.0000	0.0000	0.0000

POX: peroxidase; SOD: superoxide dismutase; CAT: catalase; PPO: polyphenol oxidase; LOX: lipoxygenase; PAL: phenylalanine ammonia lyase; CHI: chitinase; GLU: β 1,3 glucanase
